# Biodiversity and ITS-RFLP Characterisation of *Aspergillus* Section *Nigri* Isolates in Grapes from Four Traditional Grape-Producing Areas in Greece

**DOI:** 10.1371/journal.pone.0093923

**Published:** 2014-04-07

**Authors:** Dimosthenis Kizis, Pantelis Natskoulis, George-John E. Nychas, Efstathios Z. Panagou

**Affiliations:** Laboratory of Microbiology and Biotechnology of Foods, Department of Food Science and Human Nutrition, Agricultural University of Athens, Athens, Greece; Ecole des Mines d'Alès, France

## Abstract

A study on the occurrence of *Aspergillus* section *Nigri* species on grapes from four traditional grape-producing areas in Greece during the 2011/2012 vintage, and their capability to produce OTA was conducted. One hundred and twenty-eight black aspergilli isolates were characterised at the species level initially by the use of morphological criteria in accordance with appropriate keys, followed by molecular characterisation performed with Polymerase Chain Reaction–Restriction Fragment Length Polymorphism (PCR-RFLP) of the 5.8 ribosomal RNA gene Internal Transcribed Spacer region (5.8 rRNA ITS). Restriction enzyme digestion of the ITS amplicons using the *Hha*I, *Hinf*I and *Rsa*I, endonucleases distinguished eleven different patterns of restriction fragment length polymorphism (RFLP), four for each of the *Hha*I and *Rsa*I digests and three for *Hinf*I. From a total number of 128 individual isolates, 124 were classified into four *Aspergillus* species corresponding to *A. carbonarius, A. tubingensis, A. japonicus* and *A. ibericus*, and the remaining 4 were classified as members of the *A. niger* aggregate. *A. carbonarius* and *A. tubingensis* being the main representative species were equally counted, with higher geographical representation of the former in southern and the latter in northern regions, respectively. All isolates were tested for their ochratoxigenic potential by use of High Performance Liquid Chromatography (HPLC) and Enzyme Linked Immuno Sorbent Assay (ELISA), resulting in significant interspecies differences in OTA production.

## Introduction

Ochratoxin A (OTA) is a naturally occurring mycotoxin, produced principally by a wide range of *Aspergillus* and *Penicillium* species [1] while various nephrotoxic, carcinogenic, immunotoxic, genotoxic and teratogenic effects [2], [3], [4], [5] as well as Balkan Endemic Nephropathy [6], [7] have been attributed to this mycotoxin.

OTA is receiving attention due to its high incidence in a wide range of food commodities such as cereal-based products, coffee, spices, nuts, olives and grape-derived products [8], [9], [10], [11], [12], [13]. RASFF Annual Report places OTA as the mycotoxin with most notifications for fruit and vegetables in 2012, while is being always second after aflatoxins in notifications on mycotoxins for food and feed for over the last decade [14]. Several publications report the high occurrence of OTA in wine and grape products from different European countries, with higher OTA levels reported for products originating from southern European regions and in particular in regions with a Mediterranean climate [15], [16], [17], [18], [19], [20]. Since 2005 the European Commission has imposed regulatory limits for OTA, and has established a 2 *μ*g l^−1^ maximum level of OTA in wine and grape products [21], [22].

Several studies performed worldwide have shown that OTA is produced during infection of grapes in vineyards mainly by mycotoxigenic strains of black aspergilli (section *Nigri*), in particular *Aspergillus carbonarius* and species belonging to the *Aspergillus niger* aggregate such as *Aspergillus niger* and *Aspergillus tubingensis*, [18], [19], [20], [23], [24], [25]. *A. carbonarius* although less common than other black aspergilli, is considered to be the predominant species responsible for OTA contamination in grapes and wine, because of the ability of almost all of its strains to produce high levels of the toxin [26], [27]. Due to the variations in the toxin production potency of different aspergilli, it is of great importance to identify the *Aspergillus* species accurately in order to define potential toxicological risks at an early stage [28].

Black aspergilli are difficult to classify following only morphological criteria [29]. Though *A. carbonarius* can be easily recognized, closely related morphospecies like the biseriate species included in the *A. niger* aggregate, or uniseriate species such as *Aspergillus aculeatus* and *Aspergillus japonicus*, have been always difficult to distinguish. Among the molecular approaches used to decipher the *Aspergillus* taxonomy [30], [31], [32], [33], [34], [35], [36], [37], [38], [39], Polymerase Chain Reaction - Restriction Fragment Length Polymorphism (PCR-RFLP) has been used successfully to identify *Aspergillus* species [33], [39]. The non-coding Internal Transcribed Spacer regions (ITS) due to their high degree of sequence polymorphism have been used widely for fungi molecular systematics at the species level [40], [41], [42]. Standard primer pairs have been used for selective amplification of these fungal sequences and have been proved useful for the identification of *Aspergillus* species [33], [39].

The objective of this study was to characterize (i) the biodiversity of black aspergilli isolates from vineyards of four traditional grape producing regions in Greece during the 2011/2012 vintage using molecular techniques and (ii) study their ochratoxigenic potential. To our knowledge this is the first study with the objective to monitor the population of black aspergilli by molecular means in Greece.

## Materials and Methods

### Ethics Statement

Part of this study was carried out in private lands. No specific permissions were required for these locations/activities that took place and all the land owners agreed to perform this research in their site. The field studies did not involve endangered or protected species.

### Sampling and culturing conditions

Thirty-three vineyards, located at four traditional grape-producing regions of Greece, were chosen for sampling during the 2012 harvesting period (from August to October). The sampled vineyards were located in Iraklion prefecture of Crete (n = 8), Mesogeia province of Attica (n = 11), Corinthia and Arcadia prefectures of Peloponnese (n = 7), Thessaloniki, Imathia, Florina and Pella prefectures of Macedonia (n = 7). Selection of sampling areas was made with provision to represent the whole range of the typical climatic conditions of Greece, i.e. the Peloponnese from Southern Greece and Attica from Central Greece, having both a landlocked Mediterranean climate with vineyards of high and low altitudes, respectively, Crete from the Southern Aegean Sea with insular Mediterranean climate, and Macedonia from Northern Greece having a typical mountainous climate with vineyards of high altitude.

From every vineyard five plants were selected along two major diagonal transects and two bunches were collected from each plant, resulting in a total of 10 bunches per sampled vineyard. Bunches were kept separate in sterile plastic bags and stored in portable refrigerators during transfer to the laboratory where the analysis took place within 24 hours from sampling.

From each bunch five healthy berries were randomly selected and placed directly on the surface of Dichloran Rose Bengal Chloramphenicol (DRBC) medium (LabM, UK). The plates were incubated in the dark at 25°C for 7 days.

### Mycoflora enumeration and black aspergilli isolation and identification

After incubation, incidence of infected with black aspergilli berries and distribution of most dominant grape mycoflora at genus level, were recorded. A representative number from the black aspergilli from every vineyard were isolated from DRBC and sub-cultured on Malt Extract Agar (MEA, LabM) and Czapek Dox Agar (CD, Oxoid) for further identification at species level. The initial identification of the different strains of *Aspergillus* section *Nigri* was performed using macroscopic and microscopic morphological criteria in accordance with appropriate keys [29], [43], [44]. The reference strains of *A. carbonarius*, *A. niger*, *A. tubingensis*, *A. ochraceus* and *A. westerdijkiae* were kindly provided by Prof. N. Magan from the Mycology Group of Cranfield University. The isolates were preserved at −80°C in the culture collection of the Department of Food Science and Human Nutrition of the Agricultural University of Athens, Greece.

### DNA extraction

For DNA extraction all strains were grown in Yeast Extract Sucrose broth (YES; contained per litre 20 g yeast extract and 150 g sucrose) at 30°C for 2 days. Mycelia were collected, washed briefly with ethanol 96%, and dried using Whatman No. 1 filter paper. Approximately 200 mg of mycelia from each individual strain were frozen in liquid nitrogen and ground to a fine powder. DNA extractions were performed using the NucleoSpin Plant DNA kit (Macherey-Nagel, Germany) according to the manufacturer's instructions.

### PCR reactions and Restriction Endonuclease DNA digestions

The 5.8 S-ITS region was amplified by PCR using universal primers ITS 1 and ITS 4 [45]. PCR reactions were performed in a 50 *μ*L final volume, containing 1× standard reaction buffer (New England Biolabs, UK), 2.0 mM MgCl_2_, 300 *μ*M dNTPs (each), 300 nM primers (each), 100 ng DNA template and 1.25 U of Taq DNA polymerase (New England Biolabs, UK). The PCR reactions were performed in a MJ Research PTC-200 thermal cycler (Bio-Rad Laboratories, USA), starting with an initial denaturation step at 95°C for 5 min, followed by 37 cycles consisting of 30 sec at 95°C, 30 sec at 52°C and 40 sec at 72°C, and a final extension step at 72°C for 10 min. PCR products were digested with the *Hha*I, *Hinf*I and *Rsa*I (New England Biolabs, UK) restriction endonucleases. Digestions were performed at 37°C for 3 h, in a 20 *μ*L reaction volume containing 2 *μ*L of 10X reaction buffer, 10 *μ*L amplicon, 1.5 U restriction endonuclease, and 0.2 *μ*L BSA (10 μg *μ*L^−1^) for *Hha*I digestions. PCR amplicons and their restriction digestion fragments were separated by electrophoresis at 100 V, 1×TAE buffer, in 1% and 3% agarose gels, respectively. Agarose gels were subsequently stained in ethidium bromide solution (0.5 mg ml^−1^), and DNA bands were visualized under Ultra Violet (UV) light using a Gel Doc XR^+^ system (Bio-Rad Laboratories, USA). Molecular sizes were estimated by comparison with the DNA standard GeneRuler 50 bp and 100 bp DNA ladders (Thermo Scientific, USA).

### Sequencing and Phylogenetic Analysis

PCR products were purified using a QIAquick PCR purification kit (Qiagen, USA). Sequencing was performed for both strands using primers ITS1 and ITS4 with the BigDye Terminator v3.1 cycle sequencing kit (Life Technologies, USA) in an ABI3730 xl Genetic Analyzer (Applied Biosystems, Life Technologies, USA) automatic DNA sequencer (Cemia, Greece).

Alignment of the 5.8S-ITS region sequences was performed using the CLUSTALΩ multiple sequence alignment program available at ΕΒΙ (EMBL, UK). The following *Aspergillus* spp. 5.8S-ITS sequences: FJ450778, *A. acidus*; KF815051, *A. aculeatinus*; AJ279997, *A. aculeatus*; AM087614, *A. awamori*; AJ280010, *A. brasiliensis*; AF459734, *A. carbonarius*; FJ491684, *A. coreanus*; FJ629326, *A. costaricaensis*; AY656631, *A. ellipticus*; KC796400, *A. eucalypticola*; HE818074, *A. fijiensis*; AY373850, *A. foetidus*; AJ280013, *A. heteromorphus*; FJ629334, *A. homomorphus*; AY656624, *A. ibericus*; FJ629335, *A. japonicus*; FJ629336, *A. lacticoffeatus*; KC796401, *A. neoniger*; AJ223852, *A. niger*; FJ629352, *A. piperis*; EU159216, *A. sclerotiicarbonarius*; HM853552 *A. saccharolyticus*; FJ629353 *A. sclerotioniger*; FJ629367, *A. tubingensis*; AM745757, *A. uvarumn*; FJ629368, *A. vadensis* and FR733805, *A. violaceofuscus*, were included as outgroups for the phylogenetic analysis. Genetic distances were calculated using the Jukes-Cantor, parameter model and the phylogenetic inference was obtained by the Neighbour-Joining (NJ) method [46]. The NJ tree and the statistical confidence of a particular group of sequences in the tree, evaluated by bootstrap test (1000 pseudoreplicates), were performed using the computer program MEGA version 3.0 [47].

### OTA extraction

All 128 black aspergilli isolates were centre inoculated to CYA medium (which contained per litre: K_2_HPO, 1 g; Czapek concentrate, 10 ml; trace metal solution, 1 ml; yeast extract, 5 g; sucrose, 30 g; agar, 15 g) [41] and incubated at 25°C for 7 days in order to assess their ochratoxigenic potential. *Aspergillus* section *Nigri* isolates was subjected to Ochratoxin A determination according to the Bragulat et al. protocol [48] with a slight modification in the OTA extraction step. Instead of removing 3 small agar plugs, the whole content of the Petri dish was removed and used for OTA extraction [49], [50]. Specifically, the content of the Petri dish (substrate and mycelium) was weighted in order to express OTA production per g of substrate and extraction took place with 100 ml of an 80/20 methanol/water solution of HPLC grade purity when prepared for HPLC analysis and a 50/50 solution when prepared for the ELISA method. The weighted substrates were blended with the solutions for 2 min and left for a total of 30 min before filtered, at first through a Whatman No 1 filter paper, and subsequently through Millex nylon membrane filter of 0.2 *μ*m pore size (EMD Millipore Corp. Billerica, USA), and kept at −80°C until analysis. Additionally, known concentrations of OTA (50, 100 & 500 ppb) were spiked on CYA and recovery rates for both HPLC and ELISA methods were estimated, resulting in satisfactory recovery percentages of 96–99% and 84–95% for the former and latter method, respectively.

### HPLC Analysis

Ochratoxin A analysis was performed using reverse-phase High Performance Liquid Chromatography with fluorometric detection (HPLC-FLD). This consisted of a JASCO AS-2055Plus autosampler, a JASCO LC-Net II/ADC system controller, a JASCO PU-980/LC-980-02 pump, and a JASCO FP-2020Plus fluorescence detector (JASCO Inc., Easton, USA). The samples were separated using a C18 analytical column (250×4.6 nm, 4 *μ*m, Resteck Co., Pinnacle II, Bellefonte, USA) under isocratic conditions at a flow rate of 1 ml min^−1^ of the mobile phase (water/acetonitrile/acetic acid: 99/99/2). All chemicals used for HPLC analysis were HPLC grade (methanol and acetic acid: Sigma-Aldrich Co., Germany; water and acetonitrile: Carlo Erba Reactifs SDS, Val de Ruill, France). An excitation wavelength of 333 nm and an emission wavelength at 460 nm were used for UV detection. Standard solutions were made from stock ochratoxin A solution (10.06 *μ*g ml^−1^ in acetonitrile; Biopure, Romer Labs Diagnostics GmbH, Tulln, Austria) in mobile phase and a recovery study took place by spiking known concentration solutions to substrate and following the same extraction procedure as for samples. Run time for samples was 14 min with OTA being detected at about 11 min. The limit of quantification was 2.0 ng OTA g^−1^ CYA (ppb), while the limit of detection was 1.0 ng OTA g^−1^ CYA.

### ELISA

Ochratoxin A quantification was additionally performed with a competitive direct enzyme-linked immunosorbent assay (CD ELISA). Veratox ELISA quantitative test kit (Neogen Corp. Ltd, Lansing, USA) was applied to the samples, having a detection limit of 1 ppb (ng g^−1^) and range of quantification of 2–25 ppb. ELISA was strictly performed with accordance to the manufacturer's protocol and optical densities were determined at 650 nm absorbance with a spectrophotometer (Synergy HT, Biotek, USA). Quantification of OTA concentration was estimated with the aid of standard curves obtained from standard solutions provided within the test kit. When the determination of OTA by HPLC revealed concentrations greater than the upper limit of quantification by the ELISA method, serial dilutions of the extracts with the extraction solution took place in order to accomplish the ELISA method without extrapolation of its standard curves. Finally, non-ochratoxigenic isolates were omitted from screening with the ELISA method.

## Results

### Diversity of grapes' fungal population and *Aspergillus* section *Nigri* presence during the harvesting period

The genera of filamentous fungi most abundant on sampled grapes were, in descending order, *Aspergillus* (39%), *Fusarium* (15%), *Alternaria* (12%), *Cladosporium* (11%), *Rhizopus* (8%), *Penicillium* (7%) and *Botrytis* spp. (5)%. With respect to the different regions studied, *Aspergillus* was the most frequently isolated species in all regions (32%–55%) with the exception of Macedonia in Northern Greece where *Alternaria* was dominant (36%).

Among the potential OTA-producing fungi, only black aspergilli (*Aspergillus* section *Nigri* group) were isolated for further identification to species level. Initially, the 128 different black aspergilli species isolated from grapes were classified according to their morphological characteristics into three subgroups namely, *Aspergillus niger* aggregate, uniseriate species and *A. carbonarius*, and thereafter, a partial molecular identification took place.

**Figure 1:**
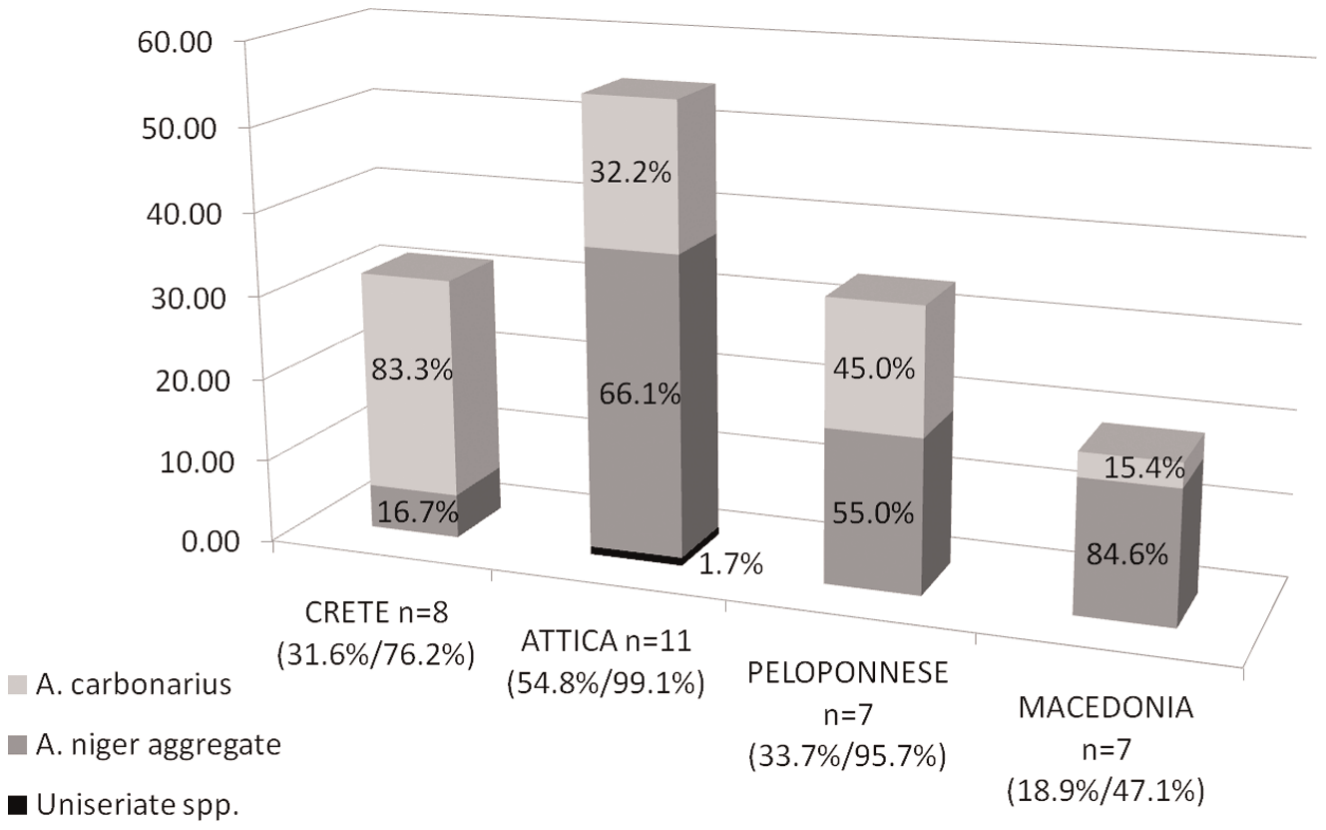
*Aspergillus* section *Nigri* strains distribution. *A*. section *Nigri* distribution in total mycoflora (bars) and different species distribution within the *A*. section *Nigri* group (percentages inside bars) for each sampled prefecture. The percentages underneath prefectures' names refer to distribution and incidence of *A*. section *Nigri*.

Incidence of black aspergilli isolation from the sampled vineyards is presented in Figure 1. Regarding the presence of *Aspergillus* section *Nigri*, Macedonia presented very low numbers, namely 47% incidence (i.e., % of contaminated out of total analysed grapes) with 18% distribution in the isolated mycoflora (i.e., % of group in total recorded fungi), while between the other regions occurrence of black aspergilli was similar and always the dominant genera. The highest incidence and distribution were observed in Attica (99% incidence/55% distribution), followed by the Peloponnese (96%/34%) and Crete (76%/32%).

Regarding *A. carbonarius*, the highest distribution occurred for isolates from Crete, with 83% of black aspergilli identified, followed by the Peloponnese and Attica with 45% and 32%, respectively, while *A. carbonarius* isolation was most scarce in vineyards of Macedonia (15%). As regards uniseriate spp. a sole isolate was identified originating from Attica prefecture. According to the spore size that can discriminate between black uniseriate aspergilli, the isolate could be characterized as *A. japonicus*.

### Molecular characterization of *Aspergillus* isolates by PCR-RFLP

**Figure 2:**
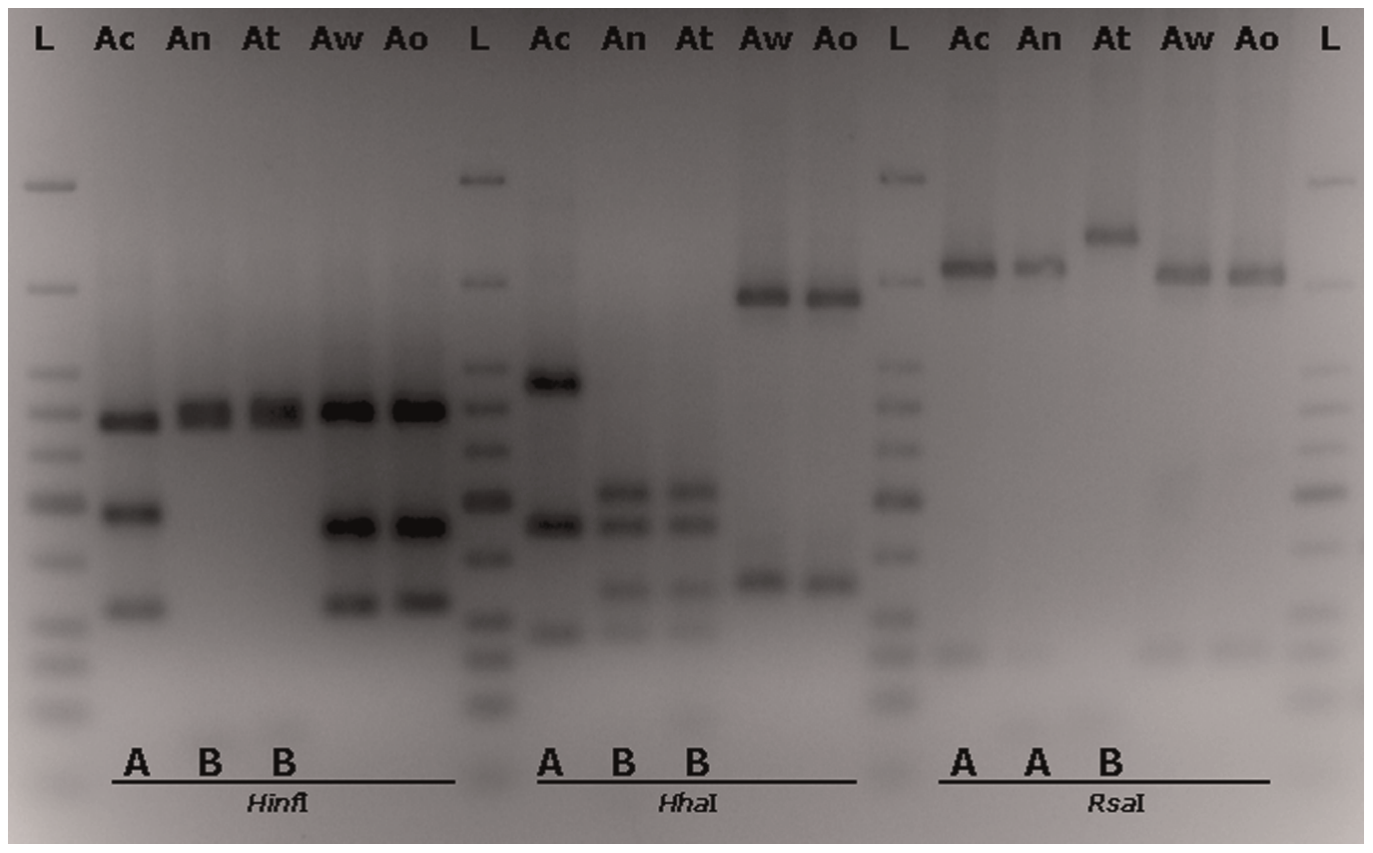
Ribosomal 5.8S-ITS region restriction digestion patterns of *Aspergillus* reference strains. Restriction digestion patterns (designated as **A** and **B**) of ribosomal 5.8S-ITS DNA amplicons from *Aspergillus* reference strains, after digestion with the restriction endonucleases ***Hinf*** **I**, ***Hha*** **I** and ***Rsa*** **I**. **Ac**: *Aspergillus carbonarius*, **An**: *Aspergillus niger*, **At**: *Aspergillus tubingensis*, **Aw**: *Aspergillus westerdijkiae*, **Ao**: *Aspergillus ochraceus*, **L**: Low molecular weight DNA ladder (molecular sizes are 766, 500, 350, 300, 250, 200, 150, 100, 75, 50 and 25 bp respectively).

**Figure 3:**
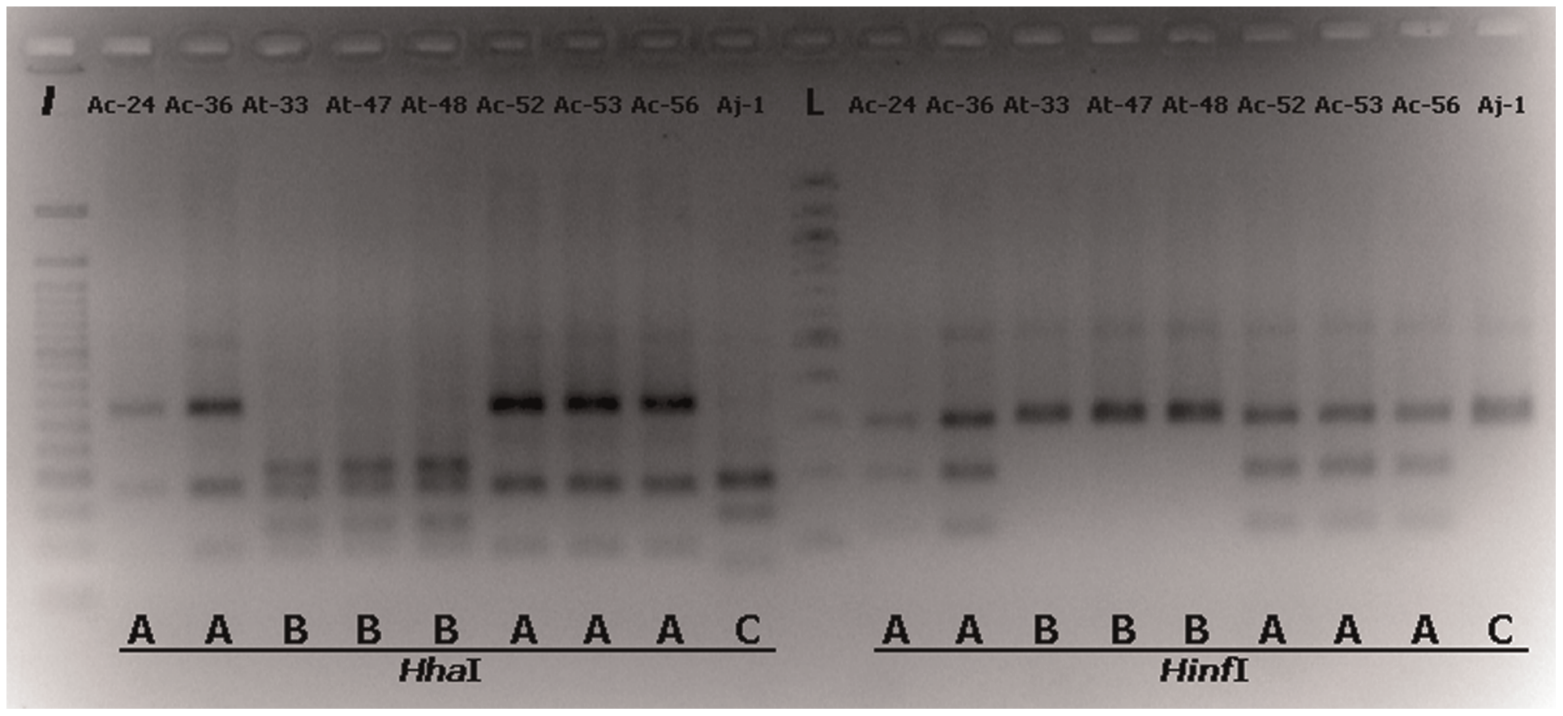
Ribosomal 5.8S-ITS region restriction digestion patterns of *Aspergillus* grape isolates. Restriction digestion patterns (designated as **A**, **B** and **C**) of ribosomal 5.8S-ITS DNA amplicons from various *Aspergilli* grape isolates (presented as isolate designations), after digestion with the restriction endonucleases ***Hha*** **I** and ***Hinf*** **I**. 50 bp **(** ***l*** **)** and 100 bp **(L)** DNA ladders are also shown.

**Figure 4:**
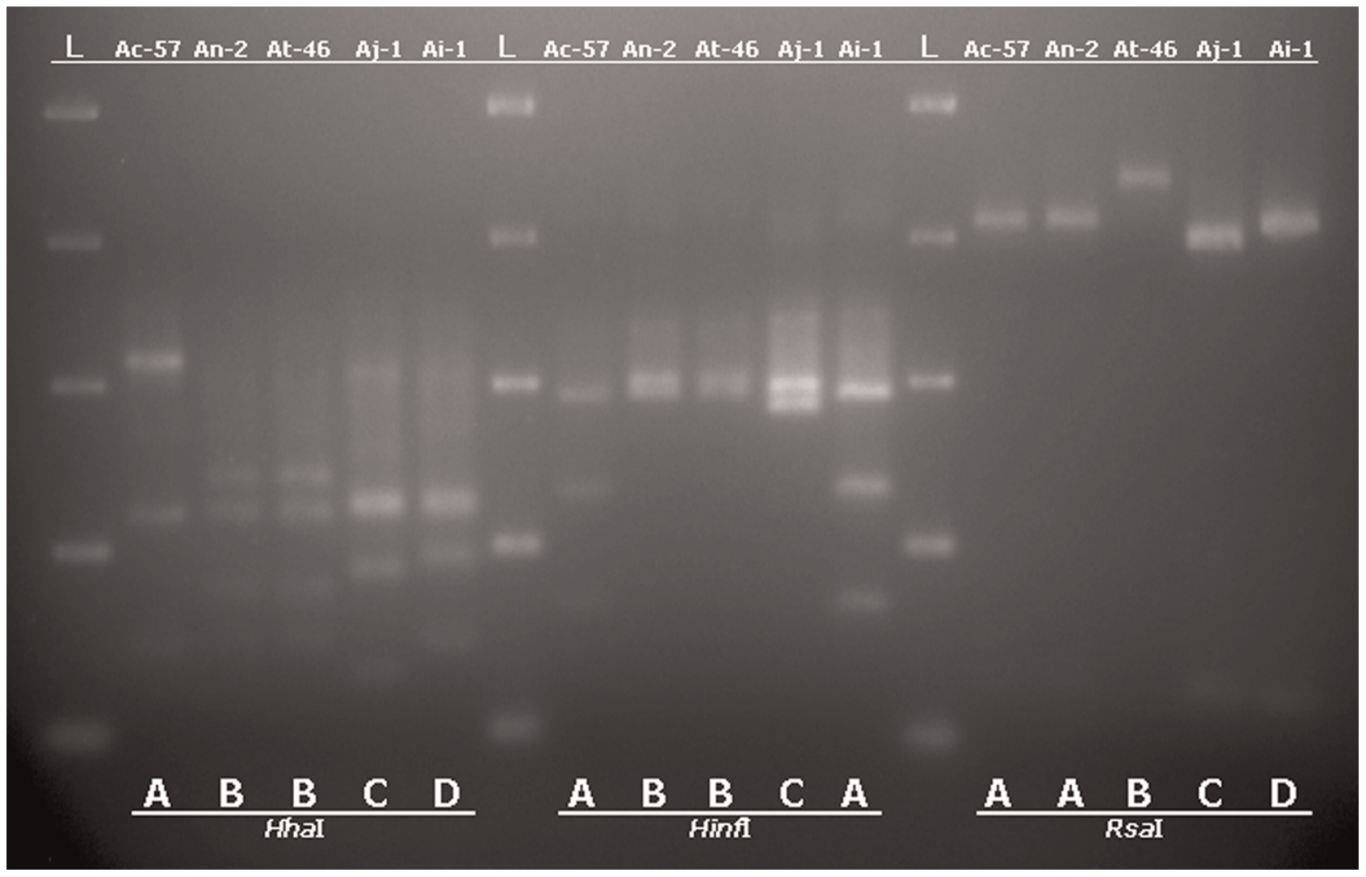
Restriction digestion patterns of sequenced ribosomal 5.8S-ITS region amplicons. Restriction digestion patterns (designated as **A**, **B**, **C** and **D**) of five sequenced ribosomal 5.8S-ITS DNA amplicons from five different *Aspergilli* grape isolates (presented as isolate designations), after digestion with the restriction endonucleases ***Hha*** **I**, ***Hinf*** **I** and ***Rsa*** **I**. Each isolate is a representative of the five different *Aspergillus* species characterized in this study. **L**: DNA ladder.

In order to monitor the representation of black aspergilli (*A. aculeatus*, *A. awamori*, *A. brasiliensis*, *A. carbonarius*, *A. foetidus*, *A. ibericus*, *A. japonicus*, *A. niger*, *A. tubingensis* and *A. uvarumn*) reported to be isolated from grapes [20], [25], [34], [37], [38] we focused on a partial molecular characterization of the 128 isolates at the species level by use of PCR-RFLP of the 5.8S-ITS region. PCR mediated amplification of the ribosomal region was performed using the ITS1 and ITS4 primers resulting to amplicon molecular sizes of approximately 600 bp for all samples. RFLP analysis was done using the *Hha*I, *Hinf*I and *Rsa*I restriction endonucleases in order to differentiate among species. The restriction patterns of reference strains as presented in Figure 2 were used for comparison with the individual isolates restriction digestion patterns. *Hha*I and *Hinf*I enzyme digestion can differentiate *A. carbonarius* from *A. niger/A. tubingensis* since they both generate 2 different restriction patterns designated here as A (for *A. carbonarius*) and B (for *A. niger* and *A. tubingensis*). *Rsa*I digestion can distinguish further *A. niger* from *A. tubingensis* generating different restriction patterns for these two species, pattern A (common for *A. niger* and *A. carbonarius*) and pattern B (for *A. tubingensis*). A total of 128 black *Aspergillus* isolates previously characterized by morphological criteria were analyzed by PCR-RFLP and their restriction patterns were compared with those obtained from the reference strains. Digestion with *Hinf*I resulted to 61 A and 66 B pattern profiles and 1 for a new profile designated as C and closely resembles profile B. *Hha*I digestion resulted to 60 A, 66 B pattern profiles and 2 for new pattern profiles designated as C and D. These new profiles for both enzymatic digestions can be seen for samples Aj-1 and Ai-1 in Figures 3 and 4. Restriction digestion with *Hha*I can also differentiate uniseriate (*A. japonicus, A. aculeatus, A. uvarum*) from all biseriate black aspergilli species [33], [39]. Depending on the pattern which will result from the *Hinf*I digestion, *A. japonicus* can be further distinguished from *A. aculeatus* [39]. According to restriction endonuclease site profile study (data not shown) of characterized black uniseriate aspergilli ITS sequences deposited in public sequence databases, *Hinf*I digestion results in a double band visible pattern for either *A. japonicus* and *A. uvarum* and a three band visible pattern for *A. aculeatus*. The 66 strains which gave a B–B pattern combination after *Hinf*I and *Hha*I digestions were subjected to digestion with the *Rsa*I, resulting to 62 B and 4 A type restriction patterns. The strain with the C-C *Hinf*I*-Hha*I, restriction digestion pattern when digested with *Rsa*I, resulted to a new profile designated as C. This profile closely resembles *Rsa*I profile A and can be seen in Figure 4. The strain with the A–D *Hinf*I*-Hha*I, restriction digestion pattern, when digested with *Rsa*I resulted to a pattern nearly indistinguishable from profile A (Figure 4). Though the molecular size of the largest DNA fragment generated (slightly above 500 bp) runs somehow faster that the corresponding band from profile A, the visible difference is not so obvious. This new profile was designated as D. According to the PCR-RFLP results for the 128 isolates, 60 (46.88%) were characterized as *A. carbonarius*, 62 (48.44%) as *A. tubingensis*, 4 (3.12%) as members of the *A. niger* aggregate, 1 (0.78%) – resembling the pattern C-C-C for *Hinf*I, *Hha*I and *Rsa*I digestions – could be characterized as *A. japonicus/A. uvarum*, and 1 (0.78%) with an A-D-D *Hinf*I, *Hha*I and *Rsa*I digestion pattern could not be clearly classified to any of the previously referred species. A detailed classification according to both phenotypical and molecular data can be seen in Table 1.

**Table 1:** Identification, origin and OTA production of *A*. section *Nigri* isolates.

Spp. - No	Species	Origin	OTA level (ppb)	
			HPLC	ELISA
At-1	*A. tubingensis*	Crete	<LOD	
At-2	*A. tubingensis*	Crete	<LOD	
Ac-1	*A. carbonarius*	Crete	31.01	32.20
Ac-2	*A. carbonarius*	Crete	27.79	43.16
Ac-3	*A. carbonarius*	Crete	42.40	44.85
Ac-4	*A. carbonarius*	Crete	1208.42	803.52
Ac-5	*A. carbonarius*	Crete	814.63	665.20
Ac-6	*A. carbonarius*	Crete	1208.79	906.39
Ac-7	*A. carbonarius*	Crete	1040.77	550.92
Ac-8	*A. carbonarius*	Crete	1290.46	590.72
Ac-9	*A. carbonarius*	Crete	1270.25	585.80
Ac-10	*A. carbonarius*	Crete	116.90	62.46
Ac-11	*A. carbonarius*	Crete	6512.18	4828.77
Ac-12	*A. carbonarius*	Crete	442.60	290.79
Ac-13	*A. carbonarius*	Crete	1013.88	405.31
Ac-14	*A. carbonarius*	Crete	1274.33	837.26
Ac-15	*A. carbonarius*	Crete	706.68	415.62
Ac-16	*A. carbonarius*	Crete	62.44	31.88
Ac-17	*A. carbonarius*	Crete	68.88	13.42
Ac-18	*A. carbonarius*	Crete	347.18	61.63
Ac-19	*A. carbonarius*	Crete	229.36	79.67
Ac-20	*A. carbonarius*	Crete	397.93	107.69
Ac-21	*A. carbonarius*	Crete	556.58	140.77
Ac-22	*A. carbonarius*	Crete	44.58	23.65
Ac-23	*A. carbonarius*	Crete	355.52	279.08
Ac-24	*A. carbonarius*	Crete	94.86	18.25
Ac-25	*A. carbonarius*	Crete	760.72	299.86
Ac-26	*A. carbonarius*	Crete	644.05	615.97
At-3	*A. tubingensis*	Crete	227.37	314.39
Ac-27	*A. carbonarius*	Crete	<LOD	
At-4	*A. tubingensis*	Crete	<LOD	
At-5	*A. tubingensis*	Crete	<LOD	
At-6	*A. tubingensis*	Crete	<LOD	
Ac-28	*A. carbonarius*	Crete	12782.62	5010.56
Ac-29	*A. carbonarius*	Crete	34825.69	30456.77
Ac-30	*A. carbonarius*	Crete	86.00	58.42
At-7	*A. tubingensis*	Attica	<LOD	
Ac-31	*A. carbonarius*	Attica	2263.18	742.60
At-8	*A. tubingensis*	Attica	<LOD	
Ac-32	*A. carbonarius*	Attica	31.68	17.79
At-9	*A. tubingensis*	Attica	<LOD	
At-10	*A. tubingensis*	Attica	<LOD	
At-11	*A. tubingensis*	Attica	<LOD	
Ac-33	*A. carbonarius*	Attica	18752.53	7221.62
Ac-34	*A. carbonarius*	Attica	748.87	216.94
At-12	*A. tubingensis*	Attica	<LOD	
At-13	*A. tubingensis*	Attica	<LOD	
At-14	*A. tubingensis*	Attica	<LOD	
Ac-35	*A. carbonarius*	Attica	553.06	341.50
At-15	*A. tubingensis*	Attica	<LOD	
Ac-36	*A. carbonarius*	Attica	121.32	116.19
An-1	*A. niger* aggr.	Attica	<LOD	
Ac-37	*A. carbonarius*	Attica	54.13	69.34
Ac-38	*A. carbonarius*	Attica	57.92	43.92
At-16	*A. tubingensis*	Attica	<LOD	
At-17	*A. tubingensis*	Attica	<LOD	
At-18	*A. tubingensis*	Attica	<LOD	
At-19	*A. tubingensis*	Attica	<LOD	
Ac-39	*A. carbonarius*	Attica	618.33	393.06
At-20	*A. tubingensis*	Attica	<LOD	
At-21	*A. tubingensis*	Attica	<LOD	
Ac-40	*A. carbonarius*	Attica	182.46	97.34
Ac-41	*A. carbonarius*	Attica	194.28	107.03
Ac-42	A. carbonarius	Attica	17.64	7.85
At-22	A. tubingensis	Attica	<LOD	
At-23	A. tubingensis	Attica	<LOD	
At-24	A. tubingensis	Attica	<LOD	
Ac-43	A. carbonarius	Attica	4.71	7.73
Ac-43	A. carbonarius	Attica	2.07	2.03
At-25	A. tubingensis	Attica	<LOD	
Ac-45	A. carbonarius	Attica	671.44	496.88
Ai-1	A. ibericus	Attica	<LOD	
At-26	A. tubingensis	Attica	<LOD	
At-27	A. tubingensis	Attica	<LOD	
Ac-46	*A. carbonarius*	Attica	4.26	3.83
An-2	*A. niger* aggr.	Attica	<LOD	
At-28	*A. tubingensis*	Attica	<LOD	
At-29	*A. tubingensis*	Attica	<LOD	
At-30	*A. tubingensis*	Attica	<LOD	
Ac-47	*A. carbonarius*	Attica	3924.93	4041.81
At-31	*A. tubingensis*	Attica	<LOD	
Ac-48	*A. carbonarius*	Attica	2725.74	2998.55
At-32	*A. tubingensis*	Attica	<LOD	
At-33	*A. tubingensis*	Attica	<LOD	
Ac-49	*A. carbonarius*	Attica	23.24	9.87
At-34	*A. tubingensis*	Attica	<LOD	
At-35	*A. tubingensis*	Attica	<LOD	
At-36	*A. tubingensis*	Attica	<LOD	
At-37	*A. tubingensis*	Attica	<LOD	
At-38	*A. tubingensis*	Attica	<LOD	
At-39	*A. tubingensis*	Attica	<LOD	
At-40	*A. tubingensis*	Attica	<LOD	
At-41	*A. tubingensis*	Attica	<LOD	
At-42	*A. tubingensis*	Attica	<LOD	
Aj-1	*A. japonicus*	Attica	<LOD	
At-43	*A. tubingensis*	Peloponnese	<LOD	
At-44	*A. tubingensis*	Peloponnese	<LOD	
At-45	*A. tubingensis*	Peloponnese	<LOD	
At-46	*A. tubingensis*	Peloponnese	<LOD	
At-47	*A. tubingensis*	Peloponnese	<LOD	
At-48	*A. tubingensis*	Peloponnese	<LOD	
Ac-50	*A. carbonarius*	Peloponnese	90.34	59.40
Ac-51	*A. carbonarius*	Peloponnese	141.51	82.34
Ac-52	*A. carbonarius*	Peloponnese	82.00	56.81
Ac-53	*A. carbonarius*	Peloponnese	65.44	55.56
An-3	*A. niger* aggr.	Peloponnese	<LOD	
Ac-54	*A. carbonarius*	Peloponnese	684.39	675.38
Ac-55	*A. carbonarius*	Peloponnese	741.88	662.32
Ac-56	*A. carbonarius*	Peloponnese	247.80	152.97
At-49	*A. tubingensis*	Peloponnese	<LOD	
At-50	*A. tubingensis*	Peloponnese	<LOD	
Ac-57	*A. carbonarius*	Peloponnese	745.14	686.79
At-51	*A. tubingensis*	Peloponnese	<LOD	
Ac-58	*A. carbonarius*	Peloponnese	167.52	116.37
At-52	*A. tubingensis*	Peloponnese	<LOD	
At-53	*A. tubingensis*	Macedonia	<LOD	
At-54	*A. tubingensis*	Macedonia	<LOD	
Ac-59	*A. carbonarius*	Macedonia	1496.38	605.75
At-55	*A. tubingensis*	Macedonia	<LOD	
At-56	*A. tubingensis*	Macedonia	<LOD	
An-4	*A. niger* aggr.	Macedonia	<LOD	
At-57	*A. tubingensis*	Macedonia	<LOD	
At-58	*A. tubingensis*	Macedonia	<LOD	
At-59	*A. tubingensis*	Macedonia	<LOD	
At-60	*A. tubingensis*	Macedonia	<LOD	
At-61	*A. tubingensis*	Macedonia	<LOD	
Ac-60	*A. carbonarius*	Macedonia	3654.06	2273.10
At-62	*A. tubingensis*	Macedonia	<LOD	

Species identification and origin of *A*. section *Nigri* spp. isolated from the present study and their ochratoxigenic potential assayed by HPLC and ELISA methods.

Limit of Quantification (LOQ) 2 ng OTA g^−1^ CYA and Limit of Detection (LOD) 1 ng OTA g^−1^ CYA.

### Sequencing and Phylogeny study

**Figure 5:**
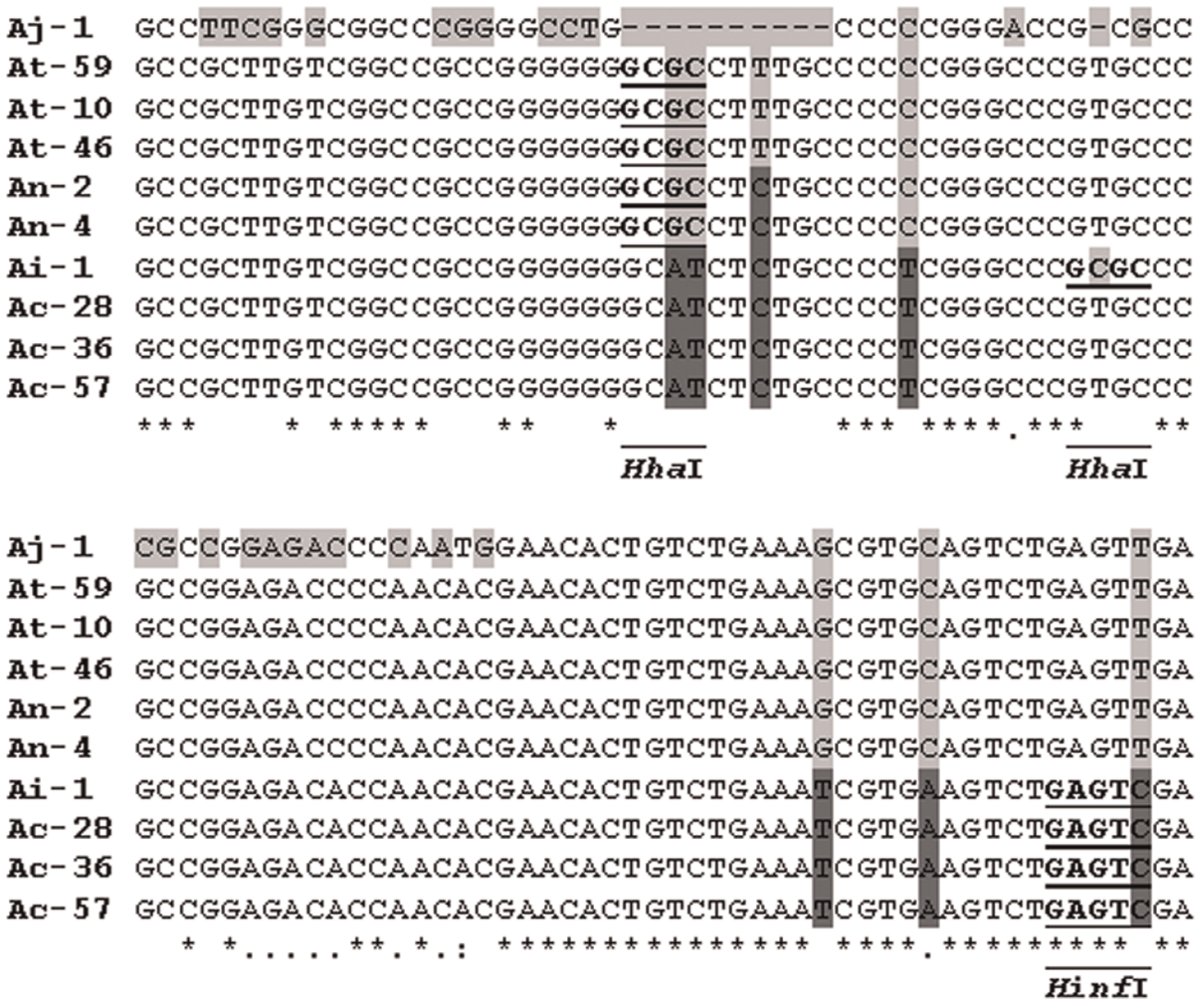
Alignment of the ribosomal 5.8S-ITS amplicon sequences of representative *Aspergilli* isolates. A part of the amplicon sequences (nucleotides 101 to 200) alignment is presented. Restriction sites are presented as bold-underlined, and variable nucleotides are highlighted. The extra *Hha*I restriction site present in the Ai-1 sequence is also shown.

Representative isolates (spp. Ac-28, At-10, Ac-36, Ai-1, An-2, Aj-1, At-46, Ac-57, An-4 and At-59) for each species as resulted from PCR-RFLP analysis were subjected to sequencing for results verification. A part from the resulted sequences alignment can be seen in Figure 5. The DNA sequence restriction pattern analysis was in accordance with these of reference strains for all isolates sequenced except isolate Ai-1. Isolate Ai-1 had a *Hinf*I restriction site pattern common to *A. carbonarius*, however, the presence of an extra *Hha*I restriction site (nucleotides 143 to 146) generated a *Hha*I restriction site profile similar to this of *A. japonicus*. A presentation of the DNA fragments generated according to the *Hinf*I, *Hha*I and *Rsa*I restriction sites, in all sequenced isolates and representative reference isolates of black aspergilli species isolated from grapes can be seen in Table 2. The fragment sizes that are generated resemble the sizes of the fragments visualized after gel electrophoresis of the amplicon digests.

**Table 2:** 5.8S-ITS RFLP DNA fragment sizes profiles.

Isolate	Rest. Endonuclease		
	*Hinf*I	*Hha*I	*Rsa*I
Ac-28, Ac-36, Ac-57 (*A. carbonarius*)	288, 191, 110, 8	330, 178, 89	521, 76
At-10, At-46, At-59 (*A. tubingensis*)	302, 289, 8	207, 178, 124, 90	599
An-2, An-4 (*A. niger aggregate*)	302, 289, 8	207, 178, 124, 90	523, 76
Ai-1 (*A. ibericus*)	289, 192, 110, 8	186, 178, 145, 90	523, 76
Aj-1 (*A. japonicus*)	293, 274, 8	185, 178, 137, 75	497, 78
*A. carbonarius* AF459734	288, 191, 110, 8	330, 178, 89	521, 76
*A. ibericus* AY656624	289, 192, 110, 8	186, 178, 145, 90	523, 76
*A. brasiliensis* AJ280010	290, 192, 110, 8	207, 178, 124, 91	524, 76
*A. niger* AJ223852	301, 290, 8	207, 178, 123, 91	524, 75
*A. tubingensis* FJ629367	302, 289, 8	207, 178, 124, 90	599
*A. awamori* AM087614	302, 289, 8	207, 178, 124, 90	523, 76
*A. foetidus* AY373850	302, 286, 8	207, 178, 124, 87	520, 76
*A. aculeatus* AJ279997	273, 183, 110, 8	185, 177, 137, 75	496, 78
*A. japonicus* FJ629335	293, 274, 8	185, 178, 137, 75	497, 78
*A. uvarum* AM745757	293, 273, 8	185, 177, 137, 75	496, 78

Individual isolates and reference strain isolates from grapes are clustered according to their RFLP profile deduced after sequencing of the 5.8S-ITS amplicon. Fragment sizes generated respect to the restriction endonuclease site position in the sequence, are given in bp.

BLAST analysis of all sequences indicated that 3 clones had high similarity to *A. carbonarius*, 3 to *A. tubingensis*, 2 to *A. niger/A. awamori*, 1 to *A. japonicus/A. aculeatus* and 1 to *A. ibericus*. The results support the characterization of the 10 isolates deduced from the RFLP analysis and suggest that the isolate with the A-D *Hinf*I-*Hha*I RFLP pattern can be classified as *A. ibericus*.

**Figure 6:**
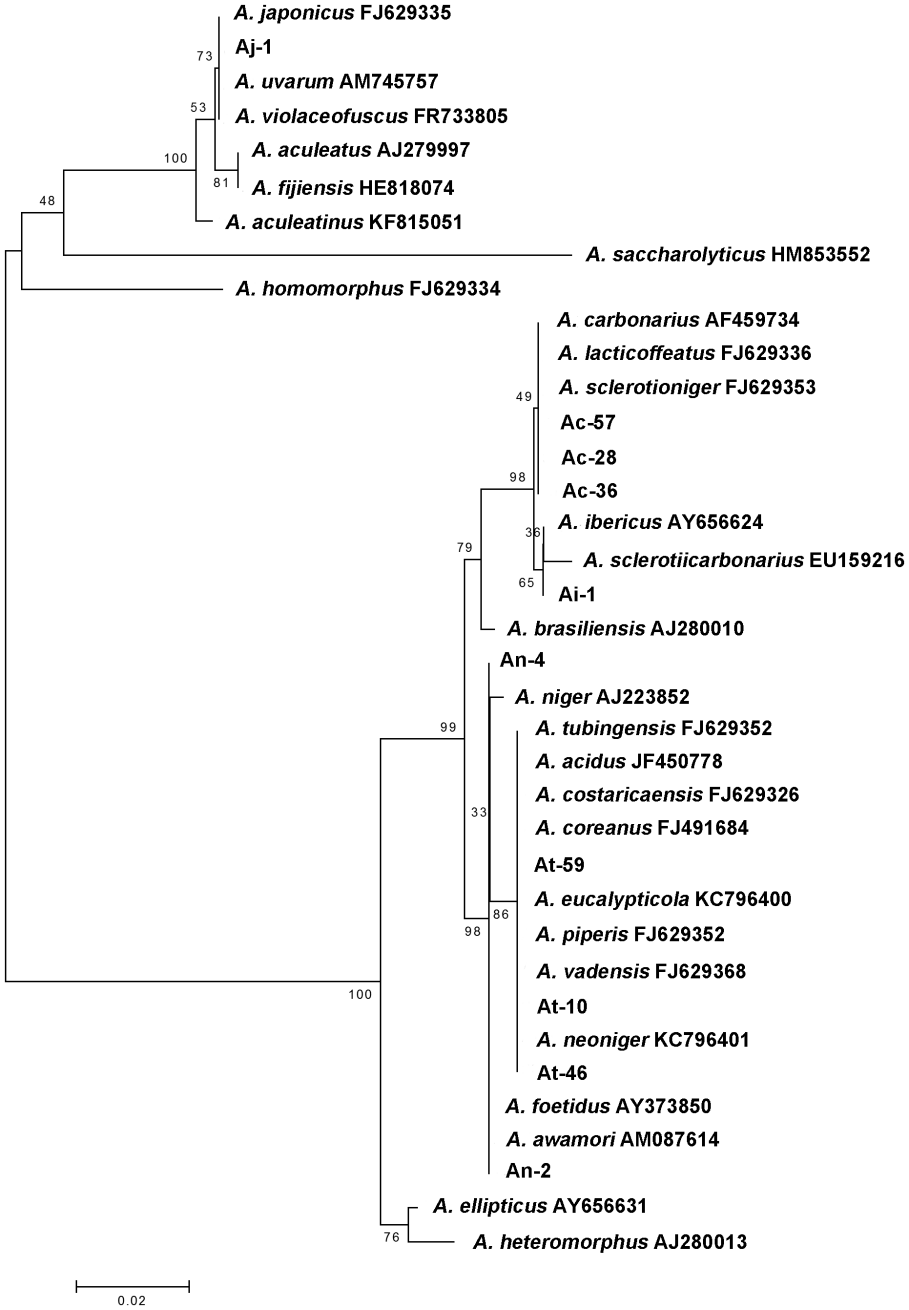
Neighbour-Joining phylogenetic tree based on divergences of ribosomal 5.8S-ITS sequences. The alignment of ribosomal 5.8S-ITS sequences from 10 isolates and 10 reference strains of *Aspergillus* spp. was performed using the CLUSTALΩ program. Nucleotide divergences were estimated according to the Jukes–Cantor model. Node numbers represent the frequency (proportion) with which a cluster appears in 1000 bootstrap runs.

The phylogenetic relationship between the isolates of *Aspergillus* species is illustrated in an N-J method cluster analysis (Figure 6). The ten sequences were aligned together with reference strains sequences retrieved from NCBI resulting to the formation of five clades. Isolate Aj-1 was aligned with uniseriate *A. japonicus, A. uvarum* and *A. aculeatus* forming together with *A. aculeatinus, A. fijiensis* and *A. violaceofuscus* a clade separated from all other biseriate *Aspergilli* sequences with a 100% bootstrap value. Two other major clades were formed. The first consisted by *A. carbonarius/A. ibericus* isolates (Ac-28, Ac-36, Ac-57 and Ai-1) together with *A. carbonarius*, *A. ibericus and A. brasiliensis* outgroup reference sequences, each one of the last three species forming a separate subgroup inside the clade. *A. carbonarius* sequences were aligned together and were separated from the *A. ibericus* sequences with a 98% bootstrap value. *A. brasiliensis* formed a separate branch in this clade with a 79% bootstrap value. The second major clade was formed by *A. tubingensis*/*A. niger* aggregate isolates (At-10, At-46, At-59 and An-2, An-4 respectively) and *A. niger* aggregate outgroup reference sequences. *A. tubingensis* sequences were clustered together with other black aspergilli (not reported to be found in grapes), forming a subgroup separated from all other *A. niger* aggregate (*A. niger*, *A. awamori* and *A. foetidus*) species of the clade, with a 86% bootstrap value. Two other minor clades were also formed, one by *A. saccharolyticus* together with *A. homomorphus* and the other by *A. ellipticus* together with *A. heteromorphus*.

### Ochratoxigenic potential of Aspergillus section Nigri species

A total of 128 isolates from grapes belonging to *Aspergillus* section *Nigri*, were tested *in vitro* for their potential to produce ochratoxin A. For the estimation of their ochratoxigenic potential a Competitive Direct ELISA procedure was also applied apart from the official HPLC method. Results showed that of the 60 *A. carbonarius* isolates 59 (98.33%) produced OTA from all geographic regions assayed. In contrast, among the *A. niger* aggregate, only 1 out of 62 isolates (1.61%), identified as *A. tubingensis*, had the potential to produce OTA. Finally, neither *A. ibericus* nor *A. japonicus* isolates were capable for OTA production.

Referring to ranges of OTA production, within the *A. carbonarius* species 1 isolate (1.67%) produced less than 1 ng OTA g^−1^ CYA, 19 isolates (31.67%) produced between 1 and 100 ng OTA g^−1^ CYA, 24 (40%) between 100 and 1000 ng OTA g^−1^ CYA, 13 (21.67%) between 1000 and 10000 ng OTA g^−1^ CYA and 3 isolates (5%) had an extremely high OTA potential with quantities greater than 10000 ng OTA g^−1^ CYA (Table 3). As regards the single *A. tubingensis* OTA producing isolate it had a potential of 227 ng OTA g^−1^ CYA.

**Table 3:** OTA ranges and distribution.

	*A. carbonarius*	*A. tubingensis*	*A. niger* aggregate	*A. ibericus*	*A. japonicus*	*A.* section *Nigri*	*A. carbonarius* OTA ranges (ppb or ng g^−1^ CYA)				
Prefecture	*n* (OTA producers)						<1	1–100	100–1000	1000–10000	>10000
Crete	30 (29)	6 (1)	0	0	0	36 (30)	1	8	11	8	2
Peloponnese	9 (9)	10 (0)	1 (0)	0	0	20 (9)	0	3	6	0	0
Attica	19 (19)	36 (0)	2 (0)	1 (0)	1 (0)	59 (19)	0	8	7	3	1
Macedonia	2 (2)	10 (0)	1 (0)	0	0	13 (2)	0	0	0	2	0
Total *n* (% ΟΤΑ producers)	60 (98.33%)	62 (1.61%)	4 (0%)	1 (0%)	1 (0%)	128 (46.88%)	1	19	24	13	3

OTA-producers and their distribution within the identified species of *A*. section *Nigri* group, and ranges of OTA potential for *A. carbonarius* isolates per prefecture.

**Figure 7:**
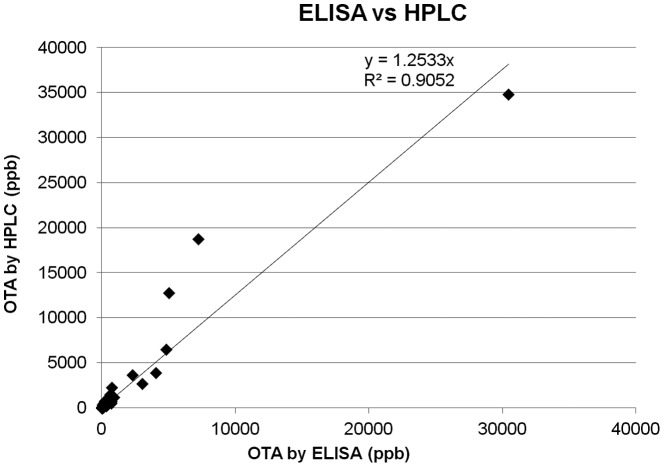
Correlation of HPLC and ELISA values for OTA determination. Linear correlation and *R^2^* for the two methods assayed for OTA quantification (ELISA & HPLC).

Regarding the performance of the ELISA test kit quantification, results were linearly correlated with those of the HPLC method with an r^2^ = 0.905 (Figure 7), but with an underestimation of the produced OTA at concentration levels above 5000 ppb. Results of both methods are presented in Table 1, together with the characterisation of the isolates after both phenotypic and molecular means.

## Discussion

Incidence of isolation and distribution of *Aspergillus* section *Nigri* group among the fungal genera were in accordance with previous reports from Greece [20], [51] and other Mediterranean countries [52], [53], [54]. A very high incidence was observed in our samples since 23 out of 33 vineyards sampled presented 100% presence for black aspergilli, although some studies have reported lower indices for vineyards from some European and South American countries [19], [55], [56], [57].

Regarding the molecular characterisation of the isolated black aspergilli, a PCR-RFLP method was applied in order to differentiate the *Aspergillus* strains. RFLP analysis of the rDNA 5.8S ITS products was performed to identify different isolates of black *Aspergillus* species from grapes compared to reference strains. A total of 128 black *Aspergilli* isolate-ITS amplicons were digested using the *Hha*I, *Hinf*I and *Rsa*I restriction digestion endonucleases resulting in 4 different RFLP patterns for *Hha*I and *Rsa*I respectively and 3 patterns for the *Hinf*I enzyme digests. In previous studies [33], [39], [58], [59], [60] according to authors' descriptions these restriction endonucleases have been used successfully to differentiate the *A. carbonarius*, *A. niger, A. tubingensis, A. japonicus and A. aculeatus* species. It was shown that *Hinf*I enzyme digestion can differentiate *A. carbonarius* from *A. niger/A. tubingensis*, and so does *Hha*I. Furthermore, *Hha*I can differentiate the uniseriate *A. japonicus*, *A. aculeatus* and *A. uvarum* species from all three previously reported biseriate aspergilli species. *Hinf*I can distinguish further *A. japonicus/A. uvarum* from *A. aculeatus* and *Rsa*I can distinguish *A. tubingensis* from all other species belonging to the *A. niger* aggregate [33], [39], [58], [59], [60].

According to the digestion pattern combinations resulted from the PCR-RFLP analysis of the 128 *Aspergilli* isolates and reference species, 60 were characterized as *A. carbonarius*, 62 as *A. tubingensis* and 4 as species belonging to the *A. niger* aggregate other than *A. tubingensis*. These results are in accordance with results presented by other authors, supporting the use of the *Hha*I, *Hinf*I and *Rsa*I restriction endonucleases for accurate characterization of *A. carbonarius*, *A. tubingensis* and, *A. niger* species [33], [39], [59], [60]. Two other digestion pattern combinations have resulted using the *Hha*I and *Hinf*I enzymes. One combination resumed a pattern presented by Spadaro *et* al. [39], suggesting that the isolate possibly belongs to the *A. japonicus/A. uvarum* species. The other digest pattern combination suggested a characterization of the isolate as *A. carbonarius* according to *Hinf*I digestion; however, this could not be supported from the *Hha*I digestion profile which closely resembled this of *A. japonicus*.

Sequencing of representative isolate DNAs supported the RFLP data since the restriction site profile of each isolate generated DNA fragments with molecular sizes that corresponded to the bands found after electrophoresis of the amplicon digests. An extra *Hha*I site is present in the sequence of isolate Ai-1 generating a restriction digestion profile different from the rest that are observed. A nucleotide substitution, C instead of T that is present in nearly all other sequences generates this extra *Hha*I site. The results presented in Table 2 show that the DNA fragments generated according to the *Hinf*I restriction sites are nearly equal in size and close to the DNA fragment sizes that generate pattern B for (*A. tubingensis* and *A. niger*). Though the pattern generated for *A. japonicus* is not identical to pattern B, it is very similar and cannot be distinguished clearly from it in a gel electrophoresis due to similar sizes and the techniques' limited resolution. A similar conclusion for unisereriate/biseriate species may be also deduced when evaluating by gel electrophoresis a profile generated after *Rsa*I digestion.

Sequence alignment with reference strain sequences retrieved from NCBI, together with BLAST analysis verified the RFLP results and showed that 3 out of the ten individual isolates were assigned to *A. carbonarius*, 3 to *A. tubingensis*, 2 showed high homology to members of the *A. niger* aggregate (*A. niger/A. awamori*) and 1 showed high homology to *A. japonicus/A. uvarum* species. Isolate Ai-1 was assigned to the species *A. ibericus* after BLAST analysis, showing 100% homology when aligned with *A. ibericus* AY656624.1, AY656623.1 and AY656622.1 strains. *A. ibericus* was first reported by Serra et al. [61] as a new *Aspergillus* species isolated from grapes and to our knowledge this is the first report that this species is isolated in Greece. Furthermore, as shown from a comparison of the *A. ibericus* 5.8S-ITS DNA sequences available at NCBI (data not shown), the fact that an extra *Hha*I site is present in all of these sequences makes it useful for characterization of *A. ibericus* by PCR-RFLP using this restriction endonuclease. The phylogenetic analysis performed using reference species' sequences as outgroups supports the sequence data and is in accordance with data presented by other authors using the ITS region for phylogenetic analysis of black aspergilli [37].

According to various authors [20], [34], [37], [38], [58], [62], [63,] black aspergilli that have been isolated from grapes fall into the biseriate *A. carbonarius*, *A. tubingensis*, *A. niger*, *A. awamori*, *A. foetidus*, *A. brasiliensis* and *A. ibericus*, and the uniseriate *A. japonicus*, *A. uvarum* and *A. aculeatus* species. Characterization of these isolates by molecular means has been based on different approximations using RFLP [25], [31], [33], [39], [64], AFLP [30], [32], [36], [65], or multilocus analysis [62], [66]. RFLP and AFLP have been used successfully for characterization of the main ochratoxigenic aspergilli isolated from grapes [25], [30], [31], [32], [33], [36], [39], [64], [65]. Multilocus analysis based on calmodulin, beta-tubulin and ITS sequences has proved a useful tool for extended characterization and comparative study of isolates from a great variety of sources such as grapes, coffee, soil, surfaces, or unknown origin [37], [62], [66]. In this work we have followed a similar approximation using ITS PCR-RFLP in order to characterize black aspergilli isolates from grapes. We have been able to characterize the isolates belonging to the *A. carbonarius*, *A. tubingensis* and *A. ibericus* species, since the RFLP patterns (Table 2) can clearly differentiate these species from all other black aspergilli species isolated from grapes.

The Aj-1 isolate could not be characterized only by molecular means since there is no difference between the RFLP patterns and ITS region DNA sequence for either species. According to the morphological observations the Aj-1 isolate was characterized as *A. japonicus* since its conidial size (4.3 μm) falls in the range of *A. japonicus* (4–5 μm) and not in the range of *A. uvarum* (3–4 μm) [34]. PCR-RFLP when used in combination with other means such as use of morphological criteria is a reliable approximation for black aspergilli characterization, where, for example, differentiation of aspergilli as biseriate/uniseriate can initially short the strains to two major groups. Isolates An-1 to An-4 have been characterized as *A. niger* aggregate members due to the limitation of the ITS PCR-RFLP to differentiate between *A. niger*, *A. awamori* and *A. foetidus* species, thus resulting to a partial molecular characterization of these four isolates.

With regard to the field study, Greece is a country with extended coast, although, areas of grape planting are characterized by different climatic characteristics. Vineyards localisation can vary from very low altitude, near seaside, either arid or wet, to very high altitudes and wet climate regions. Referring to the present study, influence of the geographic localization of the vineyards on the incidence of black aspergilli in grapes was significant. The vineyards selected for this study are representative of these different climatic profiles existing in the country. Most studies that correlated the geo-climatic conditions with the presence and distribution of black aspergilli reported a trend of higher isolation incidence in regions with higher mean temperatures, but more wet areas [19], [53], [66], [67]. These reports are partly in accordance with the present study, since the geographical localisation of sampled vineyards influenced black aspergilli presence, but with the higher incidences found in arid areas. In accordance with our results are the findings of Serra et al. [54], [56] for Spain where higher incidences for black aspergilli were reported from hot and dry regions. In addition, most of the ochratoxigenic isolates came from regions with arid climate, Crete and Attica prefecture, but this could be also correlated with the low altitude of these vineyards in contrast with the Peloponnese and Macedonia where altitudes of grape cultivation are higher. Moreover, the incidence of black aspergilli and the *A. carbonarius* isolation were much lower in Macedonia where temperatures are lower and the climate is wet compared with the other regions. In contrast, Chiotta et al. [65] have reported higher incidences of *A. carbonarius* from vineyards of higher altitudes. In addition, Perrone et al. [20] and Visconti et al. [24] denoted the strong correlation of relative humidity and rainfall with *A. carbonarius* presence and OTA accumulation in grapes. Finally, many works denote that, apart from the climate of a wider region, very important role have also the different practices used in grape cultivation and the specific microclimate of the vineyard [68], [69], [70].

*A. niger* aggregate and *A. carbonarius* were equally distributed in the present work, while only one uniseriate species was isolated and identified as *A. japonicus*. Similar distributions of *A. carbonarius* within the *A*. section *Nigri* group has been also reported by Bau et al. [69] for grapes from the Mediterranean Spanish coast and Tjamos et al. [51] for Rhodes island in Greece. These results are in accordance with our findings, since Crete, which has an insular Mediterranean climate, similar to those of Rhode and the Mediterranean Spanish coast, presented the higher incidence of *A. carbonarius* among black aspergilli. In contrast, El Khoury et al. [71] for Lebanon and Chiotta et al. [65], [66] for Argentina have reported relatively lower participation of *A. carbonarius* within black aspergilli, while Battilani et al. [19] reported very high occurrence (20%) of uniseriate species in Italian vineyards during grape ripening.

Several studies from countries all over the world during the last years have identified *A*. section *Nigri* group as the responsible fungi for OTA contamination of grapes and derived products. Specifically, *A. carbonarius* is considered as the main OTA contamination source, and to a lesser extend species of *A. niger* aggregate, mainly *A. niger* and *A. tubingensis*. This conclusion derives from the higher ochratoxigenic ability of *A. carbonarius* in contrast to *A. niger* aggregate isolates that, even when they are more abundant within the *A*. section *Nigri* group, they are either totally incapable of toxin production or their toxigenic ability is scarce and weak [19], [53], [72]. In our study, all *A. carbonarius* isolates, with one exception, were capable for OTA production with most of them having a high ochratoxigenic potential, while from *A. niger* aggregate only one isolate identified as *Aspergillus tubingensis* was capable to produce OTA *in vitro* (Table 3). Similar results have been reported for other Mediterranean countries [19], [54], [56], [69], [73], although there are reports of higher ochratoxigenic incidences among *A. niger* aggregate and uniseriate spp. [39]. Ranges of ochratoxigenic potential for *A. carbonarius* isolates varied from 0 to 34 ppm (*μ*g OTA g^−1^ CYA), with most of them producing between 1 and 1000 ppb (Table 3). Our results are also in agreement with studies contacted in Greece [51], [68], although Greek isolates with higher ochratoxigenic potential have been reported [31]. Particularly, Tjamos et al. [51], [68] in their studies have also shown that most *A. carbonarius* isolates were strong OTA producers with capabilities greater than 25 ppb, while only 6% of the *A. niger* aggregate presented high OTA potential.

## Conclusions

From our results there is a clear correlation of *A*. section *Nigri* group with the arid climate of Crete and Attica, whereas for colder and wet areas its presence is scarcer. Moreover, higher incidence of ochratoxigenic isolates and OTA production was recorded for the *A. carbonarius* isolates, confirming in this way that *A. carbonarius* is the main species responsible for OTA contamination in Greek grapes. Regarding PCR-RFLP our data indicate that *A. ibericus* can be differentiated from other aspergilli using the *Hha*I restriction endonuclease.
